# A belt for the cell: cellulosic wall thickenings and their role in morphogenesis of the 3D puzzle cells in walnut shells

**DOI:** 10.1093/jxb/erab197

**Published:** 2021-05-08

**Authors:** Sebastian J Antreich, Nannan Xiao, Jessica C Huss, Notburga Gierlinger

**Affiliations:** 1 Department of Nanobiotechnology, Institute of Biophysics, University of Natural Resources and Life Sciences, Vienna, Austria; 2 Cardiff University, UK

**Keywords:** 3D imaging, 3D puzzle cells, interlocking, Juglandaceae, morphogenesis, nutshell, primary cell wall, Raman, SBF-SEM, sclerenchyma

## Abstract

Walnut (*Juglans regia*) kernels are protected by a tough shell consisting of polylobate sclereids that interlock into a 3D puzzle. The shape transformations from isodiametric to lobed cells is well documented for 2D pavement cells, but not for 3D puzzle sclereids. Here, we study the morphogenesis of these cells by using a combination of different imaging techniques. Serial face-microtomy enabled us to reconstruct tissue growth of whole walnut fruits in 3D, and serial block face-scanning electron microscopy exposed cell shapes and their transformation in 3D during shell tissue development. In combination with Raman and fluorescence microscopy, we revealed multiple loops of cellulosic thickenings in cell walls, acting as stiff restrictions during cell growth and leading to the lobed cell shape. Our findings contribute to a better understanding of the 3D shape transformation of polylobate sclereids and the role of pectin and cellulose within this process.

## Teaser

We show the development of the 3D puzzle sclereids, which make up the whole walnut shell, and discuss the potential role of cell wall thickenings during tissue morphogenesis.

## Introduction

Fruits of the Persian walnut (*Juglans regia*) are composed of a green and fleshy husk (fused bract and bracteoles), a dry and hard shell (pericarp), and a tasty and healthy kernel protected by those two envelopes. A closer look into the shell reveals polylobate sclereid cells tightly interlocked in 3D with their neighbours, which leads to a higher contact area between cells and superior mechanical properties compared with tissues with isodiametric cells as in pine seed coats (Antreich *et al.*, 2019). Furthermore, the irregularly shaped cells are also found in shells of pecan and pistachio (Huss *et al.*, 2020).

In general, cells of plant tissues divide first and expand later during the fast growth phase of the plant organ (Gonzalez *et al.*, 2012). During expansion, hydrostatic pressure (turgor) expands the whole cell, stretches the cell wall and forces it to loosen some parts, followed by adding new materials to grow (for a review, see Cosgrove, 2018). Root and stem cells expand mainly in one axis to push the root down into the ground or the stem up into the air (Baskin, 2005; Daher *et al.*, 2018). Nevertheless, there are tissues where the cells start to expand irregularly, forming lobes as in epidermal cells of leaves (Vöfély *et al.*, 2019). The irregular shape of the cell helps to reduce mechanical stress on the cell wall caused by high turgor pressure. For example, in growing epidermal cells of *Arabidopsis thaliana*, lobes reduce the overall mechanical stresses at the cell and tissue level when cell size increases; however, high stress values become visible at the indents between the lobes (neck regions) (Sapala *et al.*, 2018).

These irregular cell shapes generate certain stress patterns which are clearly interlinked with cell wall composition and its mechanical properties (Kierzkowski *et al.*, 2019). The primary cell wall is composed mainly of polysaccharides such as cellulose, which is the main load-bearing component; pectin, which is important for cell wall flexibility; and hemicelluloses, which cross-link cellulose microfibrils (Lampugnani *et al.*, 2018). Cellulose is typically the stiffer part of the cell wall due to its crystalline structure, and the arrangement of the microfibrils is linked to the cortical microtubule distribution in the cell (Paredez *et al.*, 2006; Gutierrez *et al.*, 2009; Bidhendi and Geitmann, 2016). These cortical microtubules tend to orient along more highly stressed cell wall regions, where more cellulose microfibrils become deposited in parallel to the microtubules, thus increasing the stiffness of the cell wall (Sampathkumar *et al.*, 2014). Pectin plays a central role not only in cell–cell adhesion in the middle lamella (Marry *et al.*, 2006), but also in lobe initialization by changing the stiffness of the cell wall (Peaucelle *et al.*, 2015; Majda *et al.*, 2017; Haas *et al.*, 2020). Recently, a two-step mechanism for lobe formation has been proposed, where de-methylated pectin increases stiffness at the future indent, which leads to cell wall undulation associated with more highly stressed regions. This furthermore favours the alignment of microtubules and increased accumulation of cellulose fibrils at the indent, which slows down expansion at this location during growth (Altartouri *et al.*, 2019; Bidhendi *et al.*, 2019).

Most studies on irregular cell shapes focus on the epidermal pavement cells of *A. thaliana* or on epidermal cells of other dicotyledons, monocotyledons, and ferns (Sotiriou *et al.*, 2018; Vöfély *et al.*, 2019). In the epidermis, mainly the anticlinal walls undulate, while the periclinal walls are straight, which makes it easy to measure with confocal laser scanning microscopes in 2D. Based on that, shape descriptors are also established in 2D (Sapala *et al.*, 2018; Altartouri *et al.*, 2019; Vöfély *et al.*, 2019; Poeschl *et al.*, 2020). However, it is not known how the sclereid puzzle cells form in 3D in walnut shells.

The challenge in walnut is that the husk covers the shell tissue during fruit growth and cells in the shell expand irregularly in all directions. In this study, we uncover this morphogenesis for the first time in 3D by using a serial block face-scanning electron microscopy (SBF-SEM). Based on the 3D reconstructions, we characterize cell shapes with different shape descriptors. We also investigate the developing sclereids with Raman spectroscopy to understand the chemical contributions to lobe formation. Finally, we suggest a possible mechanism for shaping walnut puzzle sclereids in 3D.

## Materials and methods

### Sampling

We collected walnuts at 1 week intervals throughout 2019, starting from April 30th until September 30th, from the horticulture garden of BOKU, Vienna. Walnuts grew on a >40-year-old tree of the cultivar ‘Geisenheim 120’. Five nuts were always collected from the sunny side of the tree, put into plastic bags, and immediately brought to the laboratory for further investigation.

### Fresh weight, size, and serial face-microtomy

Each week the fresh weight, length, and diameter of each nut were measured. Every 2 weeks (from week 4 to 12), one of the five walnuts was used for the serial face-microtomy (SF-M). Another walnut was used for the SBF-SEM, calcofluor white staining, and Raman microscopy analysis. All other nuts were frozen at –20 °C for later use. For the SF-M, the walnut was kept in the cryostat microtome (CM3050 S, Leica Biosystems, Nussloch, Germany) for 1–4 h (depending on the nut size) at –20 °C until all liquids in the walnut were frozen. A camera was mounted in front of the walnut and after each 30–100 µm cut (depending on the walnut size) with the microtome knife a photo was taken. As the sample holder moves toward the knife, the camera position did not need changing during the cutting. The acquired picture stacks of the whole nuts were processed and registered in ImageJ (NIH, Bethesda, MD, USA) with the plugin ‘Linear stack alignment with SWIFT’ using the standard settings (Rueden *et al.*, 2017). Then the aligned stack was segmented in the Software Amira (Thermo Fisher Scientific, Waltham, MA, USA) into seed, soft shell, hard shell, and husk, followed by 3D reconstruction.

### Serial block face-scanning electron microscopy

Small pieces of walnut shell of ~1 mm×1 mm×1 mm were trimmed with a razor blade, always from the mid region of the nut close to the suture. Trimmed pieces were immersed immediately in fixation solution containing 3% glutaraldehyde in 100 mM sodium cacodylate (pH 7.4) and stored at 4 °C overnight. Samples were rinsed three times with 150 mM cacodylate buffer and post-fixed with 2% osmium tetroxide and 0.2% ruthenium red in 150 mM cacodylate buffer for 1 h at room temperature. After washing five times with cacodylate buffer, samples were incubated in freshly prepared thiocarbohydrazide solution (1% w/v in dH_2_O) for 45 min, followed by washing three times with dH_2_O and post-fixing a second time with a 2% osmium solution for 1 h. Samples were washed again four times with dH_2_O, immersed in 0.5% uranyl acetate, and stored overnight at 4 °C. Again, samples were washed five times in dH_2_O and then transferred in Waltron’s lead aspartate solution for 30 min at 65 °C, followed by washing five times in dH_2_O. Dehydration was performed in 30, 50, 70, 90, 100, and 100% ethanol in water, followed by 100% and 100% acetone; 30 min each at room temperature. Samples were then infiltrated by 25% low-viscosity resin in acetone and left at 4 °C overnight. Then samples were transferred into 50% and further into 75% resin, for 4 h each, and then in 100% resin overnight at 4 °C, followed by a second round of 100% resin for 6 h at room temperature. Samples were then embedded in flat embedding moulds and polymerized at 65 °C for 48 h. Resin blocks were trimmed with a glass knife on a UC-7 ultramicrotome (Leica Microsystems, Vienna, Austria) to 0.5 mm^3^ and glued with silver cement on a stub. They were coated with a 10 nm gold layer in an EM SCD005 sputter coater (Leica Microsystems) and mounted on the microtome of the Apreo SEM (Thermo Fisher Scientific). Scans of 100 µm^2^ were acquired with 1.18 kV, 100 pA, and 3 µs dwell time. Approximately 1000 slices with a slicing depth of 100 nm were made, controlled by the software Maps 3.4 VS (Thermo Fisher Scientific). The resulting stacks were scaled to a useable size (~1000×1000×100 pixels) for the Amira software and registered in ImageJ with the plugin ‘Linear stack alignment with SWIFT’ using the standard settings. All whole cells, which were not cut off by the border, were segmented manually. From each segmented cell surface/volume, convex hull surface/volume and contact surfaces between each neighbouring cell were calculated in the software Amira. Additionally, lobe number was calculated by the centreline tree function (tube parameter: slope 1.2, zeroVal 3.5). This made a skeleton of the cells in 3D but was very sensitive to rough cell shape. Therefore, the segmented cells were smoothed to eliminate selection artefacts, so that only main lobes were counted. Finally, the largest empty sphere (LES) of each cell was calculated with the ‘Thickness’ function of the ImageJ plugin BoneJ (Dougherty and Kunzelmann, 2007). 3D reconstruction of all cells (including cell shape descriptors) was done using Amira software (Thermo Fisher Scientific).

### Confocal laser scanning microscopy

Small pieces of shell tissue were cut out close to the suture and fixed and de-coloured according to Pasternak *et al.* (2015), with minor changes to stain cellulose with calcofluor white. Samples were put into an Eppendorf tube with 1.5 ml of pure MeOH for 20 min at 37 °C. Afterwards the sample was transferred into 0.8 ml of fresh pure MeOH for another 3 min, then 200 µl of dH_2_O was added in 2 min intervals until reaching 2 ml in total. Following this, samples were washed twice with dH_2_O for 5 min each. Samples were then transferred on a glass slide, stained with one drop of a ready-to-use calcofluor white stain solution (Sigma-Aldrich) containing 1 g l^–1^ calcofluor white M2R and 0.5 g l^–1^ Evans blue, and then mounted on a TCS SP5 II CLSM (Leica Microsystems). As emission source, a 405 nm UV diode was used, and the detection range was set from 450 nm to 500 nm. Pictures were made with the same magnification using a ×40/0.85 objective and a resolution of 0.2 µm.

### Confocal Raman microscopy

From small blocks of frozen walnut shells, 20–30 µm thin sections were cut in the cryostat microtome and transferred on a standard glass slide. Samples were washed several times with dH_2_O, followed by D_2_O, and sealed with nail polish for Raman microscopic measurements. Spectra were acquired from microsections using a confocal Raman microscope (alpha300RA, WITec, Ulm, Germany) equipped with a ×100 oil immersion objective (NA 1.4, Carl Zeiss, Jena, Germany) and a piezoelectric scan stage. A laser (λ=532 nm) was passed through a polarization-preserving single-mode optical fibre and focused through the objective with a spatial resolution of 0.3 μm on the sample. The Raman scattering signal was detected by a CCD camera (Andor DV401 BV, Belfast, UK) behind a spectrometer (600 g mm^−1^ grating, UHTS 300 WITec, Ulm, Germany). The laser power was 40 mW. For measurement set-up, the software Control Four (WITec) was used. Raman analysis was performed with Project FOUR (WITec) and Opus 7.5 software (Bruker Optik GmbH, Ettlingen, Germany). After applying cosmic ray spike removal, Raman chemical images were generated based on the integration of relevant wavenumber regions (e.g. CH stretching). The indent was selected, and a non-negative matrix factorization (NMF) was performed in Project FOUR with six basic spectra.

### Statistics

Data were analysed with the software SigmaPlot 12 (Systat Software, San Jose, CA, USA) for significant differences between each development stage. On all data from the cell segmentation, a Kruskal–Wallis one-way ANOVA on ranks was performed, followed by Dunn’s method to compare all ranks. Significant differences (*P*<0.05) are marked in the figures with an asterisk.

## Results

### Walnut and tissue growth

**Fig. 1.**
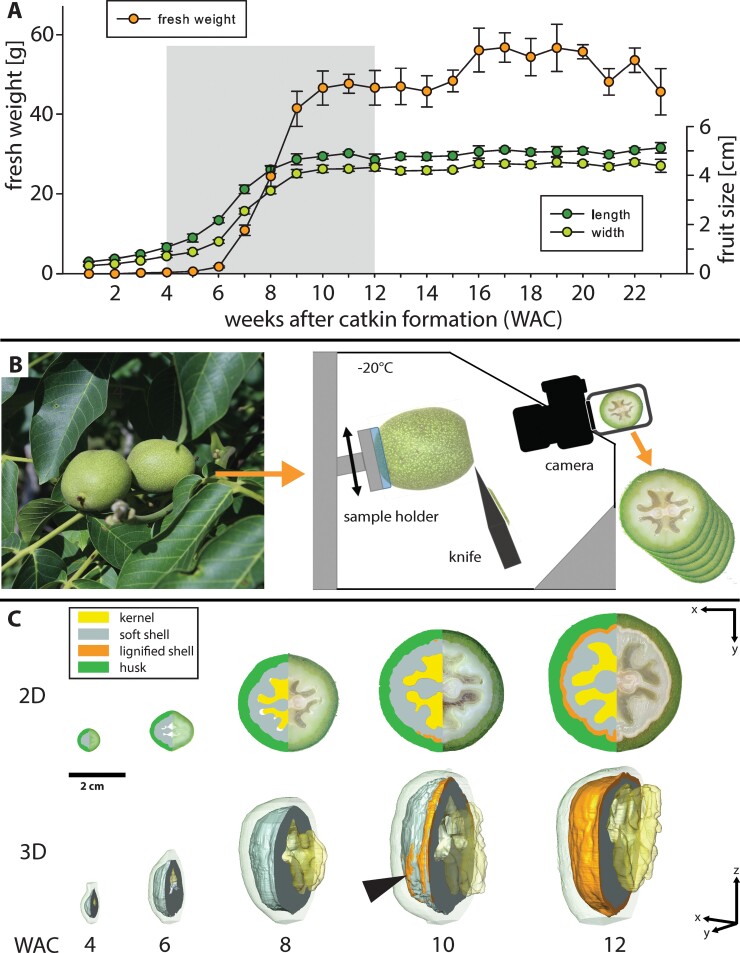
Walnut fruit development and tissue growth. (A) Fresh weight, length, and width of walnuts sampled from April 30th to September 30th in 1 week intervals corresponding to 1–23 weeks after catkin formation (WAC). The grey area shows the period chosen for serial face-microtomy (SF-M) (*n*=5, mean ±SD). (B) Freshly collected walnuts were transferred into the cryostat microtome chamber, sequentially cut, and photographed. (C) 2D segmentation and 3D reconstructions from SF-M cuts showed the development of the kernel, soft shell, lignified shell, and husk. For better visualization, only the area (2D) and the volume (3D, excluding the kernel) left of the suture are shown. Lignification (orange) started along the suture but also appeared in some areas away from the suture (arrowhead).

**Fig. 2.**
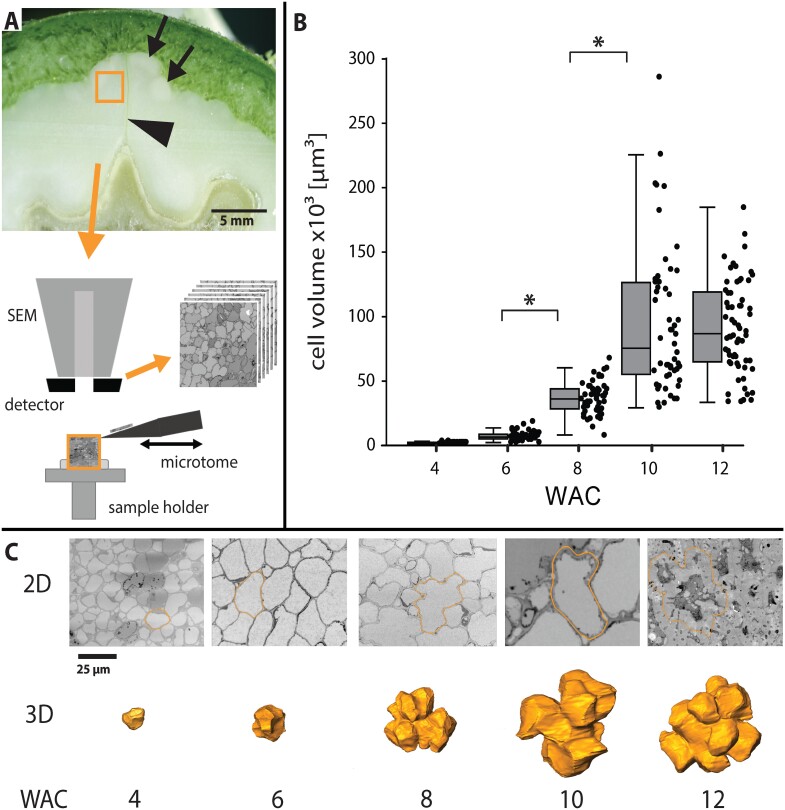
Change of cell volume and cell shape during lobe formation visualized by SBF-SEM. (A) Small pieces of shell tissue were cut out close to the suture (arrowhead), fixed, and embedded for SBF-SEM to produce serial cuts. In this cross-section of WAC 10, the shell had already started to lignify (black arrows). (B) Cell volume based on reconstructions during the growing period (box: 25–75%, whisker: 1.5 IQR, *P<0.05). (C) SBF-SEM images (2D) represent each developmental stage. The cells marked on the image had a volume closest to the average value from (B) and are shown as 3D reconstructions below (scale bar is the same for all pictures).

Our first step to track lobe formation in walnuts was a detailed monitoring of the growth and tissue development during 2019. The highest increase in weight and size occurred between 6 and 10 weeks after catkin formation (WAC), corresponding to June 3rd to July 1st, when walnut weight increased 27 times (from 1.7±0.2 g to 47±4 g) together with length and width (Fig. 1A). From WAC 4 to WAC 12, tissue development was reconstructed from picture stacks made by SF-M (Fig. 1B; Supplementary Video S1), which revealed a drastic increase of shell volume in this period (Fig. 1C). In the beginning (WAC 4–6), the kernel was only present as a small embryo, which expanded rapidly into the already formed cavity (locule) shaped by the inner part of the shell (Supplementary Fig. S1), until it filled this space at WAC 10. In the same week, the shell reached its final size and lignification started, initially along the suture from tip to base (Fig. 1C at WAC 10; Fig. 2A).

### Cell size and shape changes

During the 8 weeks of tissue growth, the cell shapes were analysed by SBF-SEM followed by 3D reconstructions (Fig. 2A, C). This detailed investigation showed a high increase in cell size during the expansion phase of the walnut (Fig. 2B). Mainly from WAC 6 to WAC 10, cell size increased 13-fold. Cell surface area expanded in the same period 8-fold (Supplementary Fig. S2). According to reconstructions of the SBF-SEM stacks (Fig. 2C), cell shape descriptors for 3D development were introduced to characterize the transition from small isodiametric cells to large polylobate cells.

**Fig. 3.**
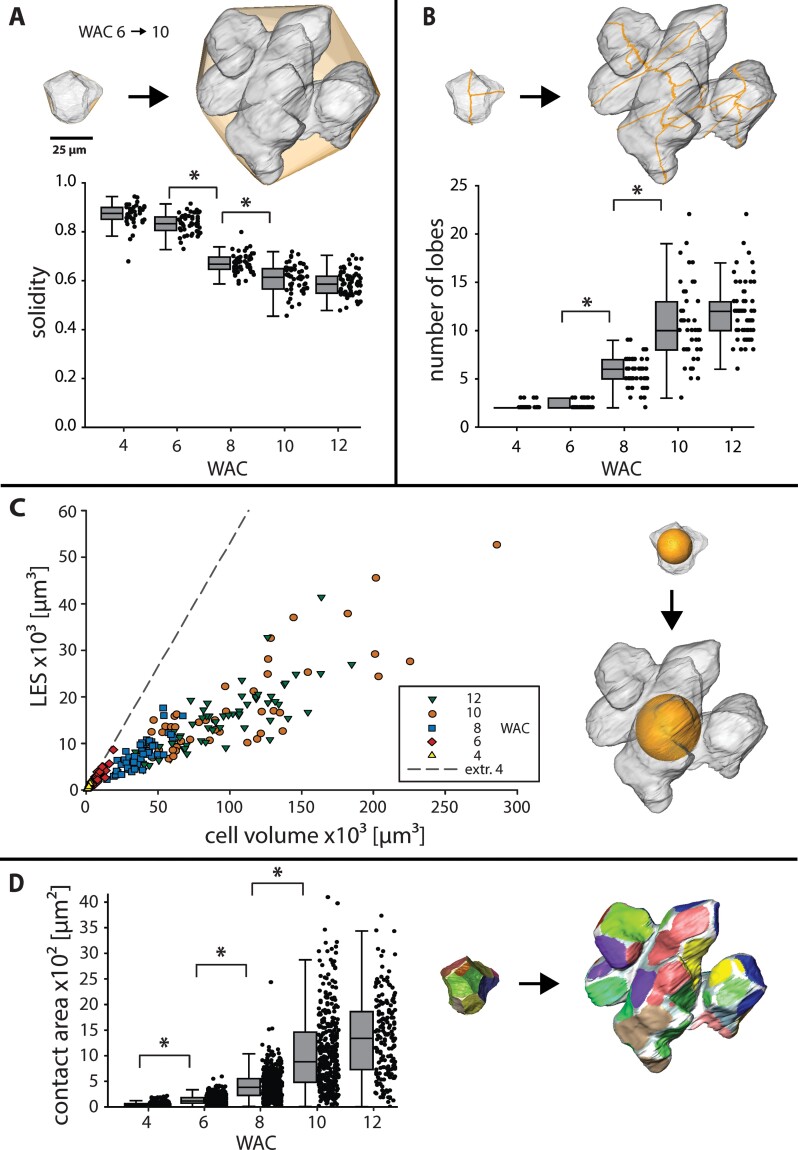
Cell shape changes in 3D during lobe formation. (A–D) Changes in cell shape descriptors from WAC 6 to WAC 10 illustrated on the same set of cells (box: 25–75%, whisker: 1.5 IQR, *P<0.05). During this interval, we could observe (A) a decrease of solidity, (B) an increase in the number of lobes after skeletonization, (C) a three times lower increase of the largest empty sphere (LES) compared with a hypothetical cell (extrapolation of WAC 4), and (D) a large increase of cell contact area of neighbouring cells and the ICS (not coloured).

Shape descriptors such as circularity (form factor), solidity, or convexity exist for 2D pavement cells of *A. thaliana* (Poeschl *et al.*, 2020). To describe the changes of the walnut cells during development, we used solidity, which represents the ratio between cell volume and convex hull volume (Fig. 3A). The solidity was 0.84±0.05 at WAC 4 and dropped to 0.61±0.06 at WAC 12.

Another tool to describe cell shape changes in 2D and 3D is the skeleton of the cell. The cell shape of the 3D model was reduced by the software to the innermost line, and the resulting skeleton endpoints corresponded to the number of main lobes (Fig. 3B). During morphogenesis, the main lobe number increased steadily from isodiametric cells (2 lobes) to polylobate cells with 12±3 lobes.

Turgor pressure causes the cell wall to bulge outwards, leading to mechanical stress on the cell wall (Cosgrove, 2018). In pavement cells of *A. thaliana*, the largest empty circle (LEC) that fits into the cells is used as a proxy for the maximal stress on the cell wall (Sapala *et al.*, 2018). However, these cells have a relative constant vertical thickness, whereas the walnut cells expand non-uniformly in all directions during growth. To extend this factor into 3D, we introduced the LES, which describes the biggest sphere that fits into the cell volume (Fig. 3C). With growing cell volume, the LES of the growing walnut cells increased around three times less by the formation of lobes compared with a hypothetical cell with similar volume but no lobes (represented by an extrapolation of the cells from WAC 4).

With decreasing solidity, the cell became more lobed, which resulted in an increase of the cell surface area per volume. Together with the fact that the number of cell neighbours stayed constant during development (Supplementary Fig. S3), cell contact area between neighbouring cells increased steadily (8-fold) from WAC 6 to WAC 10 (Fig. 3D). 3D reconstruction of single cells revealed that contact areas became separated by intercellular space (ICS), resulting in more but smaller single areas.

### Cell wall thickening and cellulose deposition

**Fig. 4.**
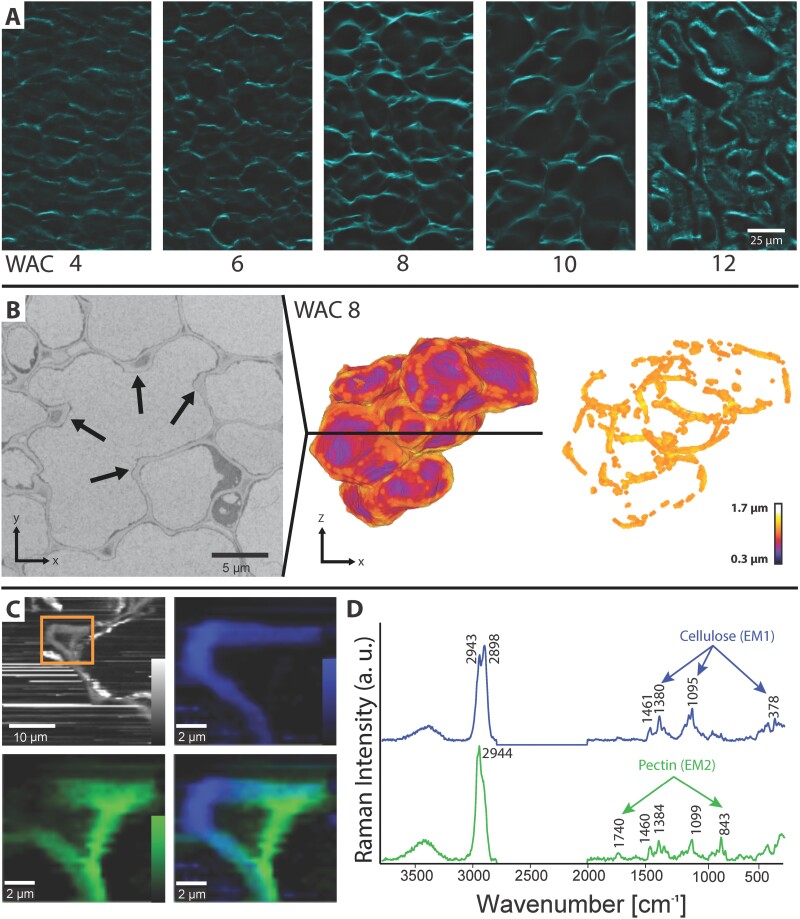
Cell wall thickening and cellulose deposition at the indents. (A) MeOH-fixed and de-coloured sections of all developmental stages after calcofluor white staining showed loops of cellulose all over the tissue at WAC 8 and WAC 10. In WAC 12, only the secondary cell wall towards the lumen is still unlignified and thus is the only part which was stained. (B) One section of the SBF-SEM stack of WAC 8 located along the black line in the 3D model. The cell showed lobes due to several indents (arrows). The cell wall was visualized based on the thickness. After removing cell wall elements, which were thinner than the average cell thickness, loops became visible. (C) Raman imaging of a section: integrating the CH stretching region from 2831 cm^−1^ to 3009 cm^−1^ revealed the organic compounds of the cell wall and deposits along the cell wall. A close up of the indent region based on non-negative matrix factorization (NMF) revealed different cell wall composition on the indent tip (blue) from that on the sides and the opposite side of the ICS (green). (D) The corresponding endmember spectra from the indents (EM1, blue) showed typical cellulose bands (1380, 1095, and 378 cm^−1^), while the green endmember (EM2) had strong marker bands of pectin at 843 cm^−1^ and 1740 cm^−1^ (for detailed analysis of the cell wall spectra, see Supplementary Fig. S5).

The changes in cellulose deposition were followed during the developmental period by staining microsections with calcofluor white (Fig. 4A). At WAC 8 and 10, loops of cellulose became visible, also seen by light microscopy before staining (Supplementary Video S2). Additionally, cell wall thickness of single cells in WAC 8 was analysed in detail in SBF-SEM reconstructions. The average thickness was 0.9±0.2 µm, with clearly thicker sites at the cell indents (Fig. 4B). By visualizing the parts which were thicker than the average cell wall thickness (values >0.9 µm), loops of thicker cell wall became visible (Supplementary Video S3). In WAC 10, the average cell wall thickness doubled (1.6±0.4 µm); the loops remained, but were less pronounced due to the thicker walls near the indents (values >1.6 µm) (Supplementary Fig. S4A; Supplementary Video S4).

To reveal the chemical composition of the native growing cell wall, Raman imaging was performed (Gierlinger, 2018). On freshly cut cross-sections from WAC 8, areas including the cell wall of the indents were scanned and, by integrating the CH stretching region from 2831 cm^−1^ to 3009 cm^−1^, all organic compounds were visualized (Fig. 4C). To elucidate details on cell wall chemistry, we cropped the region of interest (Fig. 4C, inset) and performed an NMF: an algorithm that finds the spectra of the ‘purest’ components (called endmembers, EMs) and fits the hyperspectral dataset to finally track each component within the region of interest (Prats-Mateu *et al.*, 2018). The first component (blue) was mainly found in the tips of the indents, and the second one (green) more on the sides of the ICS (where the middle lamella is located, Fig. 4C). The endmember spectrum of the first component showed bands typical for cellulose at 378, 1095, and 1380 cm^−1^ (EM1, blue spectrum), whereas the second one revealed marker bands of pectin at 843 cm^−1^ and 1740 cm^−1^ (EM2, green spectrum, Fig. 4D). The compositions of the two different cell wall spectra were modelled as a linear combination of reference spectra acquired from carbohydrates (Supplementary Table S1) using the orthogonal matching pursuit (Pati *et al.*, 1993). All three cell wall components, cellulose, hemicellulose, and pectin, were found to contribute to the cell wall spectra (Supplementary Fig. S5A, B). While hemicellulose was modelled in both spectra to a similar extent, the first one was strongly influenced by cellulose (blue, Supplementary Fig. S5A), while in the second one pectin was the main contributor (green, Supplementary Fig. S5B). So, the cell wall was more cellulosic in the tip of the indents whereas the sides of the ICS were pectin rich. From the three pectin references with different degrees of esterification (Supplementary Fig. S5C), the one with >85% esterification was chosen by the algorithm. The higher the esterification the lower the wavenumber of the marker band (856 cm^−1^ for 10–34%, 853 cm^−1^ for >85%, Supplementary Fig. S5C, inset). In the walnut cell wall spectra, the band is at an even lower position at 843 cm^−1^. On sections collected at WAC 10, higher pectin accumulation was also found at the corners of the ICS and a high cellulose signal at the indent and along the cell walls (Supplementary Fig. S4B, C).

## Discussion

Walnut fruits showed the highest increase in fresh weight between the end of May and the middle of July (WAC 4–12), which was comparable with other studies on walnut fruit development (Pinney and Polito, 1983; Drossopoulos *et al.*, 1996). Our investigation focusing on shell development during this period revealed distinct changes in cell shape—from small isodiametric cells to large polylobate cells. Between WAC 6 and WAC 10, in particular, the cells had the largest volume and surface increase and formed the lobes.

### Lobe formation of cells of walnut shell tissue

**Fig. 5.**
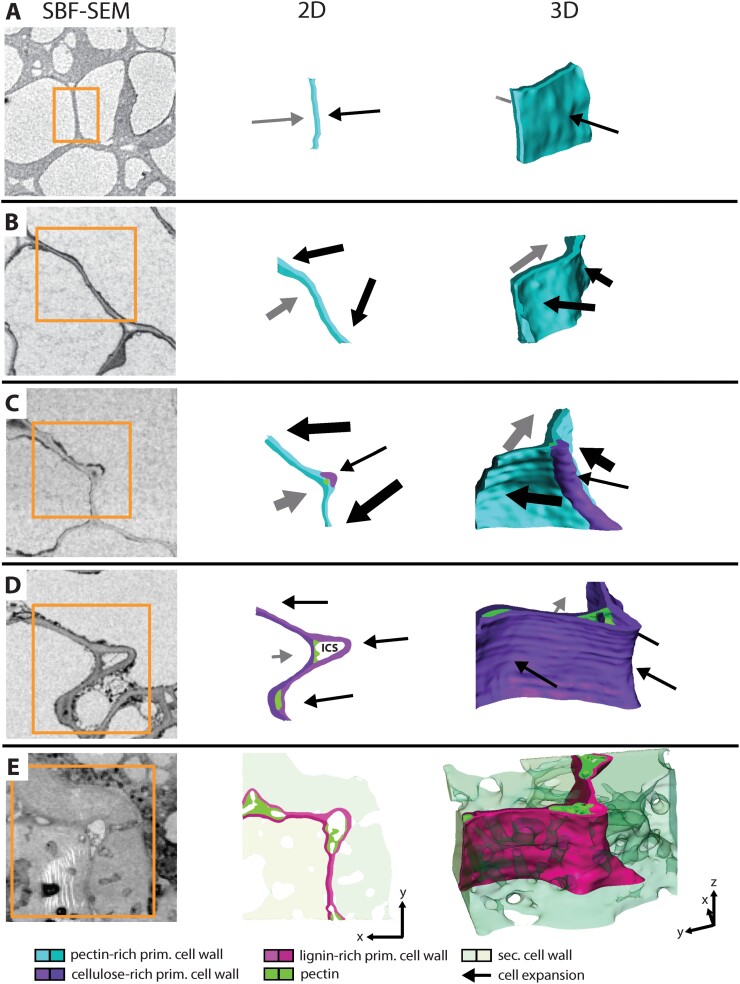
Possible scenario for lobe formation of cells in walnut shell tissue. (A–E) Representative sections showed the cell wall of neighbouring cells in each developmental stage from SBF-SEM (same scaling), a 2D sketch, and a 3D visualization of the same area. (A) After cell division, the cell wall was straight and cell expansion should be still weak (thickness of arrows represents expansion speed and direction). (B) The cell expanded further, and the cell wall started to undulate, causing different expansion directions of the neighbouring cells. (C) Due to higher cell wall stresses at the curved section of the right cell, cellulose was deposited and the thickness increased at this location. The cell expanded further but less strongly at this position, and an indent started to form. A small intercellular space (ICS) is created, first filled completely. The opposite cell expanded off-plane and formed another loop of wall thickening. (D) Later during cell growth, the ICS became bigger and showed an open space, sometimes additionally protrusions. The whole cell wall became richer in cellulose and cells expanded less. (E) Finally, secondary cell wall was deposited, and the primary cell wall was lignified, which stopped the cell expansion.

The formation of irregular cell shapes is well studied in model organisms such as *A. thaliana* (Sampathkumar *et al.*, 2014; Sapala *et al.*, 2018; Altartouri *et al.*, 2019; Bidhendi *et al.*, 2019) or *Zea mays* (Apostolakos *et al.*, 1991; Giannoutsou *et al.*, 2013). Our findings in walnuts derived from SBF-SEM and Raman spectroscopy showed features comparable with those found in *A. thaliana* during cell development (Fig. 5A–E). At the beginning of development, the cell walls between two freshly divided cells were straight (Fig. 5A). With advancing age and size, the cell wall started to undulate, which led to a wavy appearance of some contact faces to neighbouring cells (Fig. 5B). The reason for this undulation could be changes in stiffness of the cell wall or changes in pectin composition, as shown by other authors (Majda *et al.*, 2017; Altartouri *et al.*, 2019; Haas *et al.*, 2020). At the innermost part of the indent, higher stresses caused by turgor pressure most probably arose, similar to findings in the epidermal cells of *A. thaliana* (Sapala *et al.*, 2018). Presumably to counteract these stresses, cellulose was deposited along the future indents to thicken the wall—a process that is known to be mediated by cortical microtubules (Paredez *et al.*, 2006) and that we associate with the formation of the observed loops of cellulose (Figs 4B, 5C; Supplementary Video S5). These cellulosic thickenings are likely to hinder expansion at the formed indents and promote the expansion of the cell toward neighbouring cell corners. The difference in expansion caused by thicker walled indents was measured by Elsner *et al.* (2018) in *A. thaliana*, where it was found that tip regions of indents expand more slowly than the side regions.

In our study, we assumed that the restriction of expansion was so strong that cell–cell contacts were lost at the indents. SBF-SEM stacks revealed dark stained materials before the ICS was formed (Fig. 4B). Iwai *et al.* (1999) showed in his study with carrot callus that ruthenium red forms a highly electron-dense product with de-methylesterified pectins in the cell corners. Later, when the ICS opened completely and the contact with the adjacent cell wall was lost, this dark stained material was visible at the edges close to the middle lamella (Fig. 5D). At the same locations, Raman images revealed higher concentrations of pectin (Fig. 4C). Based on the band at 1740 cm^−1^ [assigned to C=O stretching of COOH (Synytsya *et al.*, 2003)], the low position of the pectin marker band at 843 cm^−1^ (Fig. 4D), and the best match with the highly esterified pectin reference (Supplementary Fig. S5C), we conclude that this was pectin with high esterification of the side groups. Bichara *et al.* (2016) reported the band at 837 cm^−1^ in citrus peel with a degree of esterification of 76%, and Synytsya *et al.* (2003) showed the band to decrease with methylation (minimum 850 cm^−1^) and increase with acetylation (maximum 862 cm^−1^). In general, pectin not only holds the cells together via the middle lamella but also controls the separation of cells (Daher and Braybrook, 2015) and, at cell corners and along the ICS in particular, turgor-mediated forces are highest (Jarvis, 1998). Other studies showed that at these locations high amounts of the highly de-methylesterified homogalacturonan are present and increase the viscosity of the cell wall matrix via Ca^2+^ bridges and delimit cell wall separation and ICS formation (Knox *et al.*, 1990; Parker *et al.*, 2001; Giannoutsou *et al.*, 2013; Sotiriou *et al.*, 2018).

However, in walnut, the stiff restrictions and the ongoing cell expansion formed a new ICS all along the cells, which is more analogous to mesophyll tissue of *Z. mays* (Giannoutsou *et al.*, 2013) or *Vigna sinensis* (Sotiriou *et al.*, 2016) than to epidermal tissue, where cell–cell contact is continuous (Sotiriou *et al.*, 2018). In mesophyll cells of *Z. mays*, cellulose deposition is parallel to the orientation of cortical microtubules, which form ring-like thickenings around the whole cell perpendicular to the leaf axis (Apostolakos *et al.*, 1991). It is shown that during tissue expansion, cells become lobed due to cellulose depositions, and the resulting ICS becomes continuously bigger. The same mechanism for lobe formation could be proposed for cells of the walnut shell, but, in contrast to *Z. mays*, the loops of cell wall thickenings were not orientated but randomly distributed. Therefore, each individual cell shaped and was shaped by other cells when they expanded into new ICS between cells, where the walls exhibited less resistance. This probably led to the observed variability of cell shapes in the shell tissue and the network-like appearance of the ICS (Supplementary Fig. S6). As development proceeded, cellulose was deposited along the whole cell wall, reducing the local variability in thickness and therefore the loops became less pronounced.

Finally, cell expansion and lobe formation ended with the onset of secondary cell wall formation and incorporation of lignin into the primary cell wall, indicated by a darker staining in SEM pictures (Fig. 5E), and was confirmed in previous studies (Antreich *et al.*, 2019; Xiao *et al.*, 2020). As lignification in walnut started along cell corners and at the net-like ICS (Xiao *et al.*, 2020), this network between the cells may play a role in facilitating the distribution of enzymes and components involved in lignification all over the shell.

### Lobed cell shape is beneficial for stress resistance on the cell and tissue level

The change from isodiametric to polylobate cells happened mainly within 4 weeks. All shape descriptors changed significantly within this period. More lobes were formed (more skeleton endpoints) and became more pronounced (reduced solidity), which led to a drastic increase in contact area to neighbouring cells. As shown in seeds of *Portulaca oleracea*, the wavy suture interface between neighbouring cells of the seed coat increases overall strength and fracture toughness compared with straight cell interfaces (Gao *et al.*, 2018). In the same way, the interlocking of the polylobate sclereid cells in walnut led to high values in tensile and compression tests on the tissue level, when compared with cells in pine seed tissue, which consists of isodiametric cells (no lobes and no net-like ICS) almost representing the shape of a tetrakaidecahedron (Antreich *et al.*, 2019; Huss *et al.*, 2020).

On the cellular level, lobed cells kept their LES low during development to reduce high stresses on the cell wall, analogous to epidermal cells in *A. thaliana* (Sapala *et al.*, 2018). So, the polylobate cell shape could have two functions: on the one hand, it reduces internal stresses on the cell wall during development; on the other hand, it increases tensile and compression strength of the whole mature shell tissue. Models derived from plant samples show that cell size and shape with its mechanical constraints influence tissue growth in 2D (Sapala *et al.*, 2018) and 3D (Bassel *et al.*, 2014). Additionally, the morphogenesis of such shell tissues is controlled *inter alia* by physical forces of the surrounding tissue (Landrein and Ingram, 2019). As shown in *A. thaliana* seeds, the pressure of the endosperm and the restriction of the seed coat affect microtubule orientation and cell wall thickening of mechanosensitive cells (Creff *et al.*, 2015; Beauzamy *et al.*, 2016). In the case of the walnut, mechanical interactions may derive from the expanding embryo and the restricting husk, forcing the area in between the cells of the shell to interlock. So, using only one cell type may simplify the coordination of growth of the tissue compared with shells with a layered arrangement of different tissues, as found for example in macadamia (Schüler *et al.*, 2014), which probably make the coordination of growth more complicated. Under these assumptions, it would be interesting to use our data to create 3D finite element models on the cellular level to shed more light on the morphogenesis of the whole walnut shell tissue.

### New insights into walnut development due to 3D visualization

SF-M and SBF-SEM are promising tools to study the morphogenesis of plant organs and tissues in 3D. In our study, SF-M is a simple and cheap tool to give insights into young and soft tissues, where X-ray computed tomography methods reach their limitation regarding the loss of contrast due to water content and loss of sharpness due to movements of the sample (Kuroki *et al.*, 2004; Kaminuma *et al.*, 2008). With samples showing differently coloured tissues, in particular, the coloured pictures unfold their full potential. In contrast, SBF-SEM gives insights into cell organization with impressively high resolution. Studies on microtubules of the mitotic spindle in human cells (Nixon *et al.*, 2017) or on endoplasmic reticulum organization in *Z. mays* (Arcalís *et al.*, 2020) are the new trend in 3D ultrastructure investigation (Smith and Starborg, 2019). Also, in this study, SBF-SEM allowed us to analyse for the first time the shape transformation of the 3D sclereid puzzle cells in walnut shell tissue. Furthermore, complex structures like the ICS network can be visualized in 3D in more detail than by using casting methods (Prat *et al.*, 1997) and this is independent of gas-filled space needed for X-ray computed tomography scans (Kuroki *et al.*, 2004). Further, SBF-SEM could be of great interest in the study of cell development in *A. thaliana* to establish life-like 3D models to better understand the role of periclinal walls in the formation of undulating cell walls (Majda *et al.*, 2017, 2019; Bidhendi *et al.*, 2019).

Finally, the combined use of state-of-the art 3D characterization and microspectroscopic methods will shed new light on still open questions, such as the stiffness differences at the beginning of cell wall undulation or the distribution of microtubules during lobe formation. Revealing the whole formation process of the 3D sclereid puzzle cells in walnut and comparing it with shells of other nuts will help us to understand the general concept of shell morphogenesis in plants.

## Supplementary data

The following supplementary data are available at *JXB* online.

Fig. S1. Visualization of the walnut kernel in the already formed cavity in 2D and 3D.

Fig. S2. Increase of cell surface from WAC 4 to WAC 12.

Fig. S3. Number of neighbouring cells during lobe formation.

Fig. S4. Raman imaging analysis of the indents at WAC 10.

Fig. S5. Modelling of the identified cell wall spectra.

Fig. S6. 3D reconstruction of the ICS around a single cell in WAC 12.

Table S1. List of reference compounds used for NMF analysis.

Video S1. SF-M picture stack and 3D reconstruction of a walnut from WAC 6.

Video S2. Cell wall thickenings in unstained tissue of walnut from WAC 8.

Video S3. 3D reconstruction of a single cell from WAC 8 showing cell thickenings along the indents.

Video S4. 3D reconstruction of a single cell from WAC 10 showing cell thickenings along the indents.

Video S5. SBF-SEM micrograph stack and 3D reconstruction of a cell wall indent from WAC 8.

erab197_suppl_Supplementary_Video_S1

erab197_suppl_Supplementary_Video_S2

erab197_suppl_Supplementary_Video_S3

erab197_suppl_Supplementary_Video_S4

erab197_suppl_Supplementary_Video_S5

erab197_suppl_Supplementary_Table_and_Figures

## Data Availability

The data supporting the findings of this study are available from the corresponding author, Sebastian J. Antreich, upon request.

## Abbreviations

EM: endmember
ICS: intercellular space
NMF: non-negative matrix factorization
SF-M: serial face-microtomy
SBF-SEM: serial block face-scanning electron microscopy
WAC: weeks after catkin formation
