# Supplementary material for: A belt for the cell: cellulosic wall thickenings and their role in morphogenesis of the 3D puzzle cells in walnut shells
Source: J Exp Bot. 2021 May 8;72(13):4744–56. doi: 10.1093/jxb/erab197 (PMC8219037; doi:10.1093/jxb/erab197)
Supplement: erab197_suppl_Supplementary_Table_and_Figures [file erab197_suppl_supplementary_table_and_figures.pdf]

Supplementary data

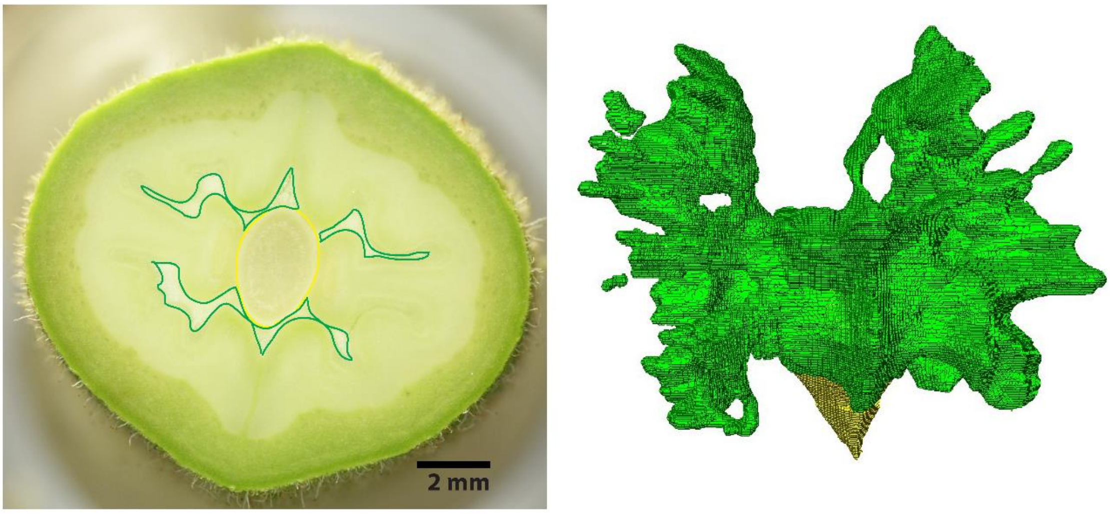

Supplementary Fig. S1. Visualisation of the walnut kernel into the already formed cavity in 2D and 3D: a) In WAC 6 the embryo of the walnut (marked yellow) exhibited only a small space of the whole cavity (green) formed by the surrounding shell tissue. **b)** 3D representation of the embryo and the formed cavity.

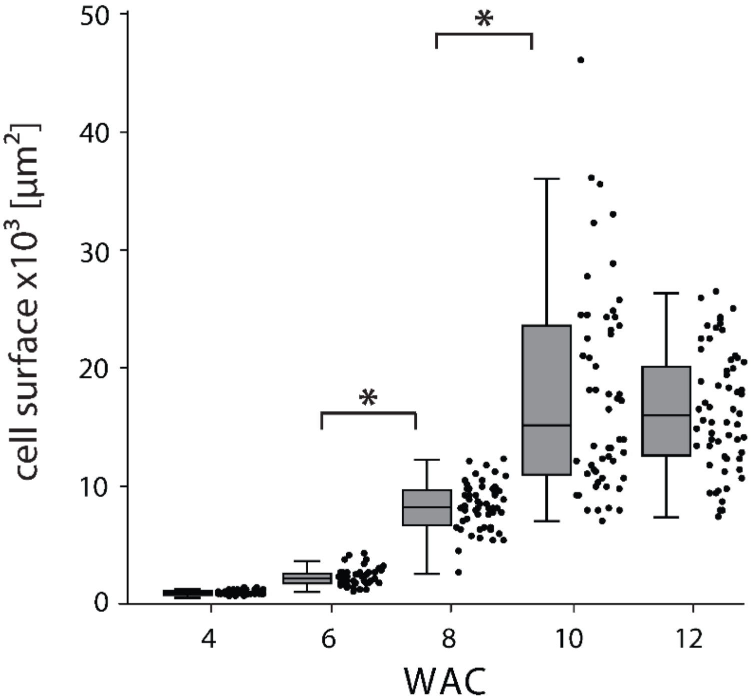

Supplementary Fig. S2. Increase of cell surface from WAC 4 to WAC 12: cell surface of at least 40 cells from WAC 4 to WAC 12. Here the same trend as in the cell volume was visible (box: 25–75%, whisker: 1.5 IQR, *P<0.05).

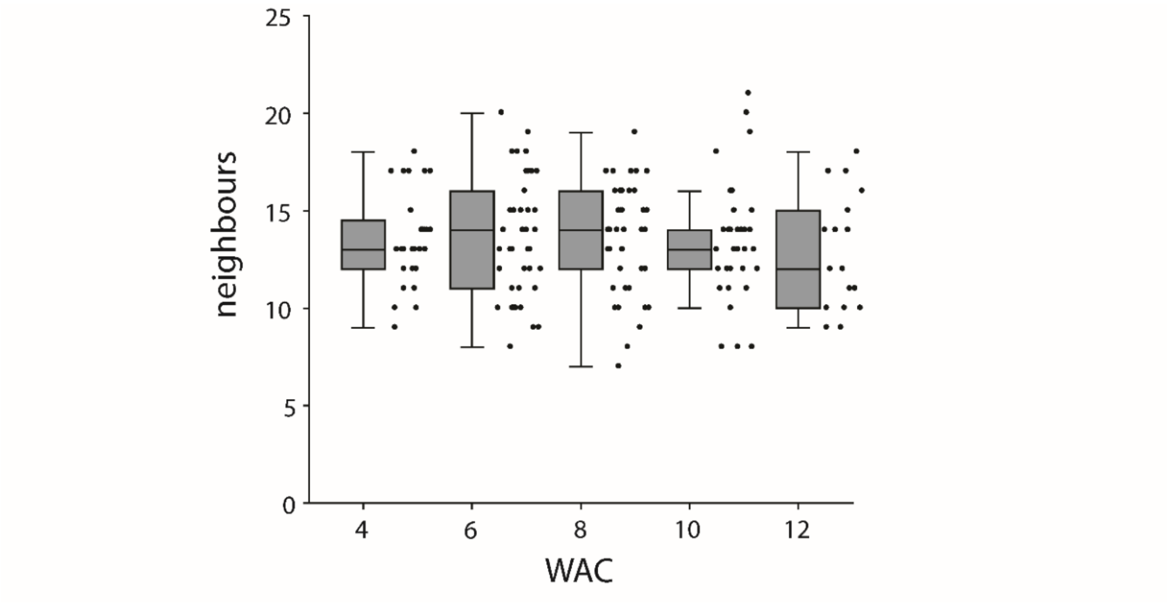

Supplementary Fig. S3. Number of cell neighbours during lobe formation: The figure shows the average number of cell neighbors counted after 3D segmentation from WAC 4 to WAC 12 (box: 25–75%, whisker: 1.5 IQR).

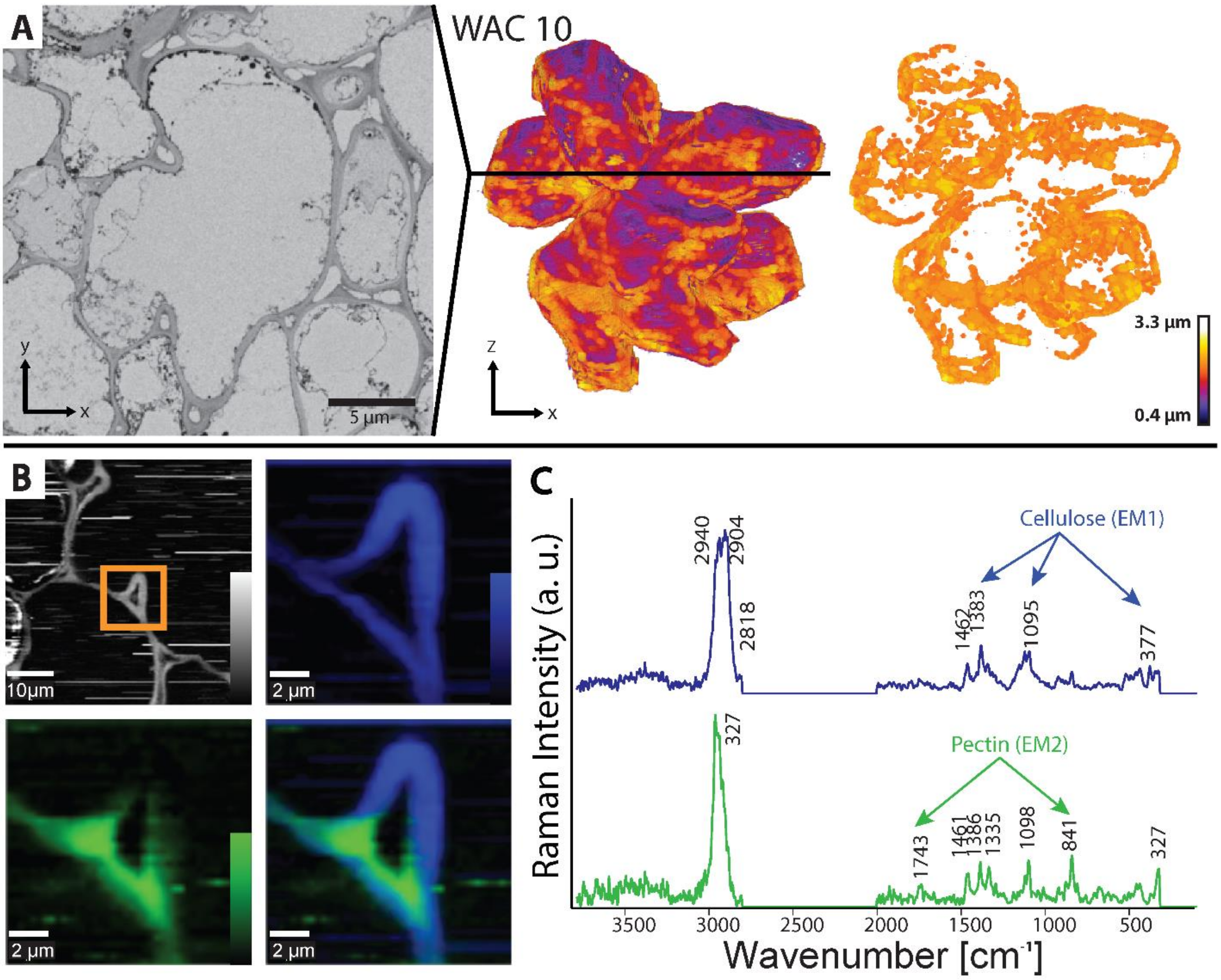

Supplementary Fig. S4. Raman imaging analysis on the indents at WAC 10: a) One section of the SBF-SEM stack from WAC 10 located along the black line in the 3D model. The cell wall was selected and the thickness was visualized. After removing cell wall elements, which were thinner than the average cell thickness less pronounced loops were visible. **b)** Raman imaging of a section integrating the CH–stretching region from 2831-3009 cm^{-1} revealed the organic material of the cell wall is uniformly distributed, surrounded by lipids. A zoom into the indent based on non-negative matrix factorization (NMF) highlighted two chemically different regions: the cell wall of the indent (blue) showed different spectral signature than the cell wall of the opposite cell and on the sides of the ICS (green). **c)** the endmember spectra revealed dominance of cellulose on the cell wall of the indent (EM1, blue) and the ICS region as pectin rich (EM2, green).

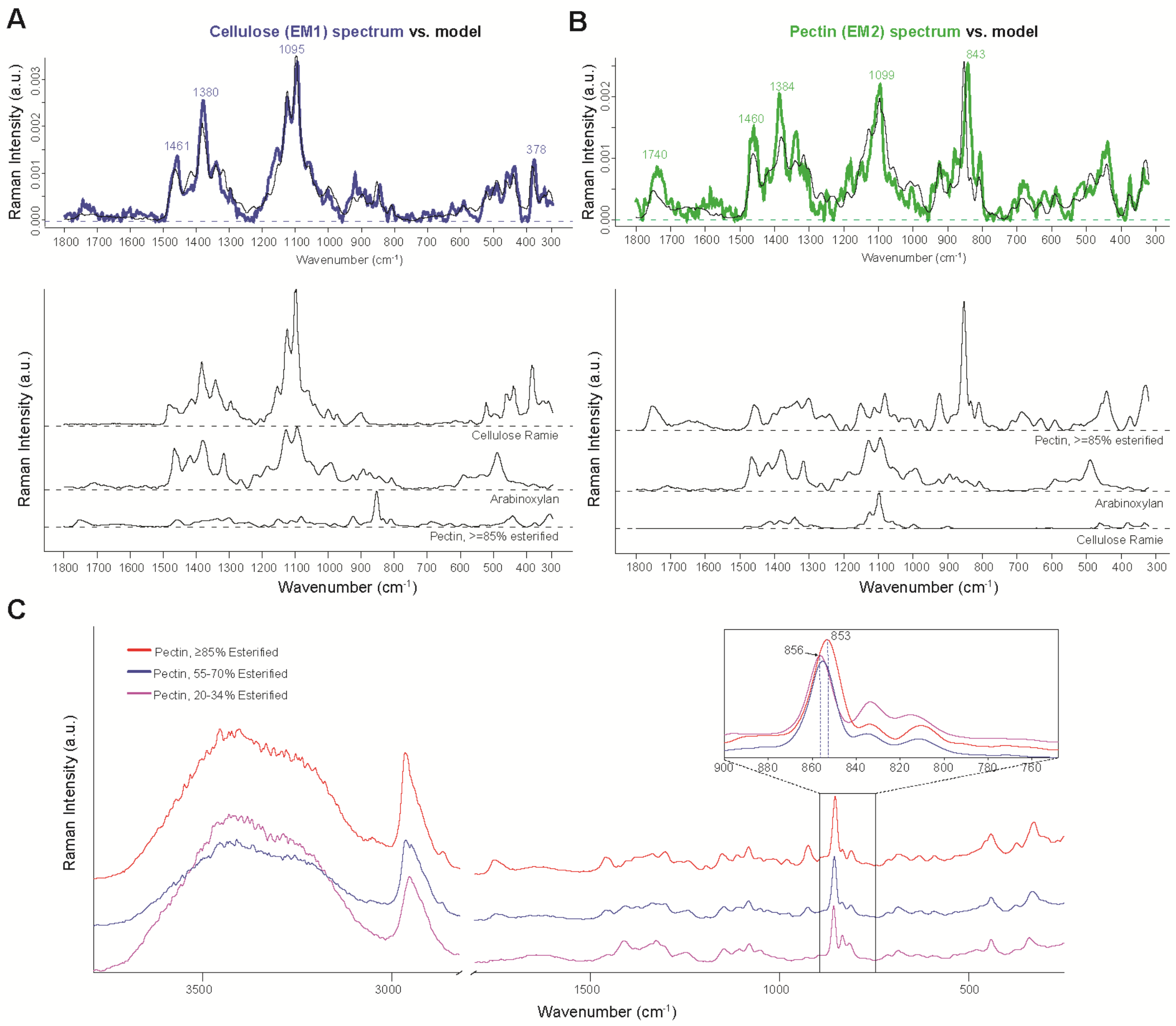

Supplementary Fig. S5. Modelling of the identified cell wall spectra: EM1 and EM 2 (Fig.4, Supplementary Fig. S4.) were modelled as a linear combination of measured reference carbohydrate spectra (see Supplementary Table S1) using the Orthogonal Matching Pursuit (Pati et al. 1993). **A)** Fitting EM1 revealed cellulose as the main component, followed by hemicellulose (arabinoxylan) and finally pectin. **B)** On contrast EM2 fitting showed pectin as the main component and cellulose in minor amounts. **C)** Reference Raman spectra of three pectins with different degrees of esterification showing the shift of the marker band from 856 to 853 cm^{-1} with increasing degree of esterification. In the models (**A**, **B**) the pectin with highest esterification was chosen to model the cell wall spectra.

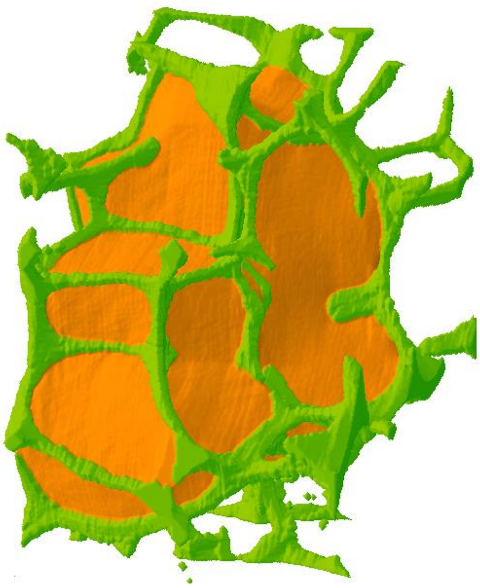

Supplementary Fig. S6. 3D reconstruction of the ICS around a single cell in WAC 12: The 3D reconstruction of the ICS (green) around single cells (orange) in WAC 12 showed a net-like shape which further was continuously distributed between the cells of the shell tissue.

Supplementary Table S1. List of reference compounds used for NMF analysis. Carbohydrate spectra from pure compounds were measured with the Raman microscope in different embedding media and used as reference spectra for linear combination.

compound	CAS	embedding medium	producer	annotation
Arabinoxylan	9040-27-1	D2O	Megazyme	from rye flour
Cellulose	9004-34-6	H2O	Dupond	
D-(+)-Galacturonic acid monohydrate	91510-62-2	D2O	Sigma	
D-(+)-Galacturonic acid monohydrate	91510-62-2	H2O	Sigma	
D-(+)-Glucose	50-99-7		Sigma	
D-(+)-Glucose	50-99-7		Sigma	
Glucomannan	11078-31-2	H2O	Megazyme	from Konjac tubers
Pectin, ≥85% esterified	37251-70-0	H2O	Sigma	from citrus fruit
Pectin, 20-34% esterified	37251-70-0	H2O	Sigma	from citrus fruit
Pectin, 55-70% esterified	37251-70-0	H2O	Sigma	from citrus fruit
Polygalacturonic acid	25990-10-7		Sigma	
Polygalacturonic acid	25990-10-7		Sigma	
Starch		H2O		from potato
Starch		H2O		from wheat
Sucrose	57-50-1	D2O		
Sucrose	57-50-1			
Xyloglucan	37294-28-3	H2O	Megazyme	
β-1,3-Glucan	9051-97-2		Sigma	
Cellulose	9004-34-6			from Ramie; laser polarization 0°
Cellulose	9004-34-6			from Ramie; laser polarization 15°
Cellulose	9004-34-6			from Ramie; laser polarization 30°
Cellulose	9004-34-6			from Ramie; laser polarization 60°
Cellulose	9004-34-6			from Ramie; laser polarization 90°

Supplementary Video S1. SF-M picture stack and 3D reconstruction of a walnut from WAC 6: Serial cut through the whole walnut fruit and 3D reconstruction of the different tissues (husk, soft shell and kernel) after segmentation.

Supplementary Video S2. Cell wall thickenings in unstained tissue of walnut from WAC 8: Light microscope stack through a tissue from WAC 8 showed loop-like cell wall thickenings. The video was made after de-coloration but before calcofluor white staining.

Supplementary Video S3. 3D reconstruction of a single cell from WAC 8 showing cell thickenings along the indents: 3D reconstruction of a single cell from WAC 8 deriving from stacks of the SBF-SEM. The cell wall was selected manually and the thickness was visualized in ImageJ. After setting the threshold for the cell wall to the average value (removing cell wall, which was thinner than the average cell thickness), loops of cell wall became visible.

Supplementary Video S4. 3D reconstruction of a single cell from WAC 10 showing cell thickenings along the indents: 3D reconstruction of a single cell from WAC 10 deriving from stacks of the SBF-SEM. The cell wall was selected manually and the thickness was visualized in ImageJ. After setting the threshold for the cell wall to the average value (removing cell wall, which was thinner than the average cell thickness), loops of cell wall became visible but were less pronounced than in WAC 8.

Supplementary Video S5. SBF-SEM micrograph stack and 3D reconstruction of a cell wall indent from WAC 8: The video shows the serial cut of a cell wall and indent between two neighboring cells from the SBF-SEM and its 3D reconstruction of the formed cell wall thickening along the indent.
